# Losses, Gains, and Changes to the Food Environment in a Rural Kentucky County during the COVID-19 Pandemic

**DOI:** 10.3390/nu13113929

**Published:** 2021-11-03

**Authors:** Makenzie L. Barr, Courtney Martin, Courtney Luecking, Kathryn Cardarelli

**Affiliations:** 1 Department of Dietetics and Human Nutrition, University of Kentucky, Lexington, KY 40506, USA; Courtney.Luecking@uky.edu; 2 Department of Biology, University of Kentucky, Lexington, KY 40506, USA; Courtney.Martin@uky.edu; 3 Department of Health, Behavior and Society, University of Kentucky, Lexington, KY 40506, USA; Kathryn.Cardarelli@uky.edu

**Keywords:** food environment, rural, appalachian, COVID-19, qualitative

## Abstract

The COVID-19 pandemic has caused alterations to be made in the way many people access, prepare, and consume food. Rural communities are particularly impacted due to pre-existing structural vulnerabilities, i.e., poverty, lack of infrastructure, and limited fresh food options. This study aimed to characterize experiences of one rural Appalachian community’s changes to the food environment during the pandemic. In April 2021, six focus groups were conducted with residents of Laurel County, Kentucky. Using grounded theory, we identified losses, gains, and overall changes to the community food environment since the onset of COVID-19. Seventeen Laurel Countians (17 female; ages 30–74) participated in the six focus groups. Three main themes emerged regarding food environment changes—(1) modifications of community food and nutrition resources, (2) expansion and utilization of online food ordering, and (3) implications of the home food environment. Rural communities faced considerable challenges during the COVID-19 pandemic, in part, due to gaps in existing infrastructure and loss of pre-existing resources. This study illustrates the complexity of changes occurring during COVID-19. Using the preliminary data obtained, we can better understand pre-existing issues in Laurel County and suggestions for future programming to address the inequitable access and response during public health emergencies and beyond.

## 1. Introduction

The COVID-19 pandemic had unprecedented consequences on the economic, political, physical, and sociocultural conditions that influence decisions about accessing, preparing, and consuming food (i.e., the food environment) [1]. Since March 2020, the onset of the COVID-19 pandemic in the United States (U.S.), economic struggles forced alterations in food behaviors such as consumption of more processed, nonperishable, low-cost foods [2] and minimizing time spent physically shopping [3,4]. Nearly 50% of U.S. households reported financial hardship, with one in five households reporting the inability to pay basic living expenses, including food [5,6]. Given the historical relationship between poverty and food security [6,7], it is not surprising that more families across the nation are experiencing hunger, with the unemployment rates surpassing all-time highs since the 1940s [6,8].

Programs such as pandemic electronic benefit transfer (P-EBT) were developed to provide aid during the declaration of public health emergencies [9]. These benefits have since increased by 15% to ensure children across the nation have sufficient food supply during the pandemic [9]. A series of three economic relief payments (stimulus checks) were also issued to provide assistance to eligible families who were impacted due to COVID-19 [10]. Though these payments, intended to provide some relief, may have assisted families in offsetting the costs of living, many were still struggling to make ends meet [11,12]. As a result, millions of Americans continued to seek supplementary meal assistance through food banks, which saw increases in the number of individuals seeking assistance for the first time [13]. Since the start of the pandemic, Feeding America reported that over 50% more individuals were utilizing food banks across the nation [13].

In addition to economic impacts on access to food, the COVID-19 pandemic disrupted food supply chains that led to alterations in supply, demand, access, and availability of foods [14]. Due to the closure of many nonessential businesses, many food access points were forced to alter operations through elements such as capacity restriction (social distancing mandates) of in-person dining rooms, reduction in money spent on dining out by patrons, limited hours of operation, and a shift to predominately take-out orders [15,16]. In response to these modifications in nonessential businesses, innovations such as eCommerce, including the availability of grocery and restaurant pick-up options to eliminate contact and increased online ordering for food delivery, changed the way individuals obtain food [17,18]. In fact, since the start of the pandemic, “food delivery” Google searches have surpassed record highs [19], likely in part, due to local public health orders to close or limit in-person dining.

Though impacts of COVID-19 are widespread [20], rural communities are among the most vulnerable with substantial impacts on employment, income, food security, and thus, well-being [21]. Pre-pandemic spatial inequalities of rural communities [22] placed these areas at further risk of consequent inequities as COVID arose. Rural areas face higher rates of unemployment and underemployment, poverty, as well as decreasing numbers of grocery stores and limited infrastructure to purchase or access emergency food supplies compared to metropolitan areas [23,24,25,26]. Since March 2020, economic and access gaps have only been exacerbated among rural communities leading to closures and pauses on many beneficial community resources [27]. However, there is a gap in understanding the downstream impact of COVID-19 on the food environment in rural communities that were structurally vulnerable before the pandemic.

Given pre-existing structural vulnerabilities [28] of rural areas such as poverty, lack of infrastructure, and limited fresh food options [29], the use of existing community resources is critical for sustaining the current state of the food environment in these rural areas [30]. In order to identify, allocate, or generate community resources that adequately support access to enough (healthy) food, the first-hand feedback and experiences of rural residents need to be heard. The goal of this study was to qualitatively examine the experiences of one rural Appalachian community in Kentucky regarding changes to the food environment during the COVID-19 pandemic. To our knowledge, this is the first qualitative study examining recent food impacts in a rural community as it results from COVID-19, and this information has the potential to influence changes in the food environment in vulnerable communities and improve preparedness for such health emergencies.

## 2. Methods

To assess adaptations of the food environment in a rural Appalachian community during the COVID-19 pandemic, community members of Laurel County, Kentucky were recruited to participate in one of six focus groups held during April of 2021. Laurel County, Kentucky, is a southeastern county with a population size of 60,813 (2021) and has 56.8% of its population classified as rural [31]. The county, which is designated Appalachian by the Appalachian Regional Commissions [32,33], is primarily white (97.0%) [34], with approximately 21.4% of people living in poverty [34], 4.4% unemployment (16.9% at its highest in April 2020 at the start of the pandemic) [35], and 17.2% are classified as food insecure [36]. Recent databases examining the COVID-19 Community Vulnerability Index highlight a county’s structural and health vulnerabilities during the pandemic. As of August 2021, Laurel County falls at a 0.69 index (of 1.00) [37], which is considered highly vulnerable. The health of Laurel County adult residents is also comparatively poor, ranking among the lower middle range of counties in Kentucky for health outcomes (such as chronic disease) and health factors (such as food environment and health behaviors) [31]. With a combined total of 20 groceries and supermarkets serving the 444 square miles of Laurel County [38], the structural vulnerability leaves community members at risk of inadequate access to quality, healthful foods. For these reasons, Laurel County was purposefully selected as a high-priority community for which to better understand healthy eating, food access, and food security, as well as related changes due to COVID-19.

This subproject was part of an overarching study developed to understand healthy eating, food access, and food security within Laurel County. During the timeframe within which this study was conducted, masks were mandated in place in public sectors, many employers continued off-site or virtual work, and school districts offered virtual learning options. As a result of pandemic mandates, two options to participate were offered (virtual participation using Zoom online conferencing platform or face to face). Community members from Laurel County, Kentucky, were invited to participate in the study through a community-engaged approach with the Kentucky Cooperative Extension Service (Extension) partners via Facebook and word of mouth. Extension is a deeply rooted, trusted entity in Laurel County. Participants self-enrolled in the study through a Qualtrics link that collected email address, age, and eligibility criteria screener questions. Participant eligibility criteria included age 18 or older; able to read, understand, and speak English; have lived in the target county for at least 1 year; and have no plans on moving out of the county within the next three years. Participants self-selected to participate in a focus group session about food and healthy eating in the county. Due to unforeseen absences and rescheduling, final groups consisted of 2 in-person focus groups that comprised 3 participants each, 2 Zoom online-based focus groups of 3 and 4 participants, and 2 mini focus groups [39] held via Zoom that contained 2 participants each (due to limited participant pool and attendance). The sample size for the 6 groups totaled *n* = 17. The Institutional Review Board at the University of Kentucky approved procedures for this study.

### 2.1. Data Collection/Measures

Individuals agreeing to participate in a focus group session were emailed their scheduled time and Zoom link or in-person meeting time and location. Informed consent was read prior to focus group sessions, and all participants verbally consented to participate. Focus group sessions were led by a single experienced trained interviewer, along with one note taker. Focus group questions from the overarching study (Table 1) asked community members to share information about healthy eating, where community members in Laurel County obtain food, and current changes in the local food environment during the COVID-19 pandemic. Questions were developed by two trained qualitative researchers and reviewed by an additional four qualitative researchers. Focus group sessions took approximately 60 min, and individuals were provided a USD 25 gift card for their time.

### 2.2. Data Analysis

Focus group sessions were recorded and transcribed verbatim by two independent coders. Multiple investigators reviewed focus group transcripts using a grounded theory approach [40]. Investigators used an iterative inductive approach to identify themes regarding changes to the food environment during the COVID-19 pandemic. These themes formed the basis of codes that were analyzed in NVivo (QSR International; Melbourne, Australia, March 2020 version). Two independent coders reviewed transcripts and assigned codes to group quotes. Coders met to discuss coding choices and discrepancies. Discrepancies were handled between the two coders and with a third, outside researcher to resolve indecisions. Codes were combined between reviewers if similar and fit under one umbrella, thus forming subthemes for each main code. Final codes and subthemes were reviewed among all authors and agreed upon. Investigators then selected illustrative quotes that represented each theme.

## 3. Results

Seventeen Laurel County adults (all female), ranging in age from 30 to 74 years (54.9 ± 12.6 SD years), participated in six focus groups. Participants described three main themes regarding how the food environment changed during the COVID-19 pandemic—(1) modifications of community food and nutrition resources, (2) expansion and utilization of online food ordering, and (3) implications of the home food environment. Table 2 provides illustrative quotes of the primary themes and subthemes that were observed. Additional quotes are provided in the File S1 in Supplementary Materials.

### 3.1. Modifications of Community Food and Nutrition Resources

Participants illuminated both losses and gains of community resources during the pandemic. Community programs and classes at the Extension office, churches, hospitals, and libraries that support healthy eating and living were paused in light of the pandemic. Participants expressed value for skill building and educational programs and classes about healthy eating, canning, and health conditions, as well as the opportunity to bring the community together. The majority of participants (i.e., more than 75%) shared negative sentiments regarding the loss of access to nutrition and health-focused classes through community agencies and hoped programs could be revived following the pandemic.

Although many health promotion programs were paused or halted because of the pandemic, participants identified the expansion of existing resources, as well as new resources, that enhanced food access in the area. Community members felt the increased demand for food assistance led to the growth in the number of sites offering food and in the quantity of food available. Local food pantries, food banks, and churches expanded efforts and offerings, along with an increase in community and personal sharing of local produce and perishable foods. Government-funded, assembled food boxes additionally offered new resources.

### 3.2. Expansion and Utilization of Online Food Ordering

Online food ordering was described as a novel resource in Laurel County. Several participants (i.e., 25–50%) highlighted new restaurant food delivery and fast-food mobile ahead ordering in the county. One participant mentioned that services such as DoorDash were unavailable due to distance, i.e., the individuals requesting DoorDash were denied because of the distance in which delivery drivers would have to travel to fulfill their request. Online ordering for curbside pick-up from local restaurants expanded during the pandemic, which seemed to be related to long drive-thru lines for one participant.

Online grocery shopping was underutilized prepandemic; however, since March 2020, more community members began using and continue to use the service. Some participants (i.e., 10–25%) felt that online grocery shopping was beneficial for healthy eating, spending less, avoiding long physical durations in store, and was a service they hope to use beyond the life of the pandemic. One participant shared a negative aspect of grocery pick-up—that grocery store employees may not choose the best quality produce as compared to if they were to personally choose their items in store.

### 3.3. Implications of the Home Food Environment

Finally, diverse experiences regarding the personal influence of the pandemic on the home food environment were shared. Participants highlighted their perceptions on the loss of employment and income negatively impacting food access among other members in the community. Reduction of income put a strain on many community members’ purchasing habits, which increased demand for the above-mentioned food assistance resources. This perception was conveyed from those volunteering for assistance programs in the community during the last year. Additionally, several participants commented on a perceived increase in community-wide utilization of fast food, with lines “wrapped around the building”.

Most participants (i.e., 50–75%) stated that as time spent at home increased as a result of the coronavirus pandemic, healthier food intake choices were made through a consequent reduction of purchasing take-out or fast-food meals. Two participants mentioned, due to closures and cancellation of events and programs, many family extracurricular activities were paused, which subsequently gave more time at home to cook and, thus, choose less convenient items out of time constraints.

For some, this increased time at home additionally led to attempts in growing their own food by gardening. While a community garden and personal gardening as a means of food intake and a hobby was stated to have increased early in the pandemic among themselves and others they know, participants noted gardening dwindled as restaurants began reopening, and the length of the COVID-19 pandemic extended. Sharing of fruits and vegetables from home gardens was identified as a benefit among this rural community.

## 4. Discussion

The COVID-19 pandemic forced adjustments to the conditions in which people make decisions about accessing, preparing, and consuming food [41]. Focus group participants from a rural county in Appalachian Kentucky highlighted positive and negative consequences on community food and nutrition resources, expansion of online food ordering services, and alterations to their own home food behaviors. Formative results highlight the next steps for Laurel County, KY, and similar rural Appalachian communities in KY [42] to include potential short-term solutions to reinvigorating the food environment as COVID-19 continues and beyond. Among the singularly studied rural Appalachian community of Laurel County, potential solutions may include restarting community education programs through various delivery modes, extending food equity throughout the community, and promoting skill building surrounding growing and cooking food at home.

Educational resources such as cooking classes and health education sessions at the Extension office, local hospital, and library are a critical connection between expertise at land grant universities or health systems and rural and underserved areas [43]. Participants in this study, who were recruited through Extension partners, confirmed the value of these types of community-based nutrition and health education resources and look forward to the return of program offerings similar to prepandemic. The pandemic required many education and healthcare organizations to pivot to virtual or remote services or programs; however, some rural residents face barriers to access to technology, internet, or cellular service, thus limiting access to services [44]. In the future, community-based nutrition and health education resources may need to be available through multiple delivery modalities to better meet people where they are. Within the current study, a major theme was the loss of these community resources and the desire for their return in some capacity. Furthermore, organizations may need support to increase capacity to deliver those types of programs.

Access to healthy food in rural areas can be complicated by the presence/location of food retail stores, transportation to food stores, and the availability and cost of food [45]. Further complicating access to adequate food in this rural county, economic conditions worsened as a result of layoffs, transitions to at-home work, and/or loss of income. Despite food safety net support from government benefits, many people in the community need additional, longer-term food assistance. As such, many nonprofit agencies work to fill the gap [46]. Participants reported community-level efforts to implement government aid for food boxes that expanded pre-existing emergency food assistance services [47] through food pantries, food banks, churches, and the community resource center. One participant shared demand for meal assistance services resulted in road closures near food distribution sites due to the number of community members who sought aid. As a valuable community resource, the continuation of these meal assistance services and aid are an area to enhance food access among this community throughout the pandemic and beyond.

For those with additional resources for food purchasing, online food/grocery ordering, restaurant delivery, and contactless grocery pick-up expanded opportunities for obtaining food. The national trend for increased availability of online food ordering [17,18] was reflected in this rural Appalachian community. Although food delivery applications may offer expansion of retail food environments to individuals living further distances, dietary quality scores of these food delivery restaurants have fallen short [48], leaving delivery as a counterintuitive new resource to supply nutritionally adequate foods (i.e., balanced, diverse, dietary pattern meeting sufficient personal energy and nutrient requirements to maintain good health) to those with limited access. Likewise, utilization of these resources can be determined by income level, access to smartphone or internet services, and delivery distance [49]. Poverty among rural dwellers adds an additional barrier to accessing food delivery options due to the expenses of delivery fees [50]. Likewise, online food ordering modalities are not typically available to those receiving Supplemental Nutrition Assistance Program (SNAP) benefits due to many factors, including both inaccessibility of benefit utilization on online platforms, as well as the lack of food access sites using online modalities for ordering as a whole [51]. As delivery became a means of expanding food access in the county during the pandemic, challenges to that access remained and may further exacerbate disparities in food access within rural communities [52]. Conducting an objective community food assessment with community representatives may be a valuable next step to comprehensively assess issues and identify resources and potential solutions that lead to a plan for community food security postpandemic [53,54].

Finally, for some, the pandemic experience encouraged healthier habits in their home food environment. National trends indicated a large shift to preparing and consuming meals at home, with 60% of consumers stating they cooked more at home as a result of the COVID-19 pandemic [55]. As the frequency of preparation and consumption of meals at home increases, dietary quality has also been shown to improve [56]. Several participants in our focus groups expressed positive sentiments that the slower pace resulting from limited commitments and paused extracurricular activities has encouraged healthier habits and benefited those with the capabilities to purchase, cook, and eat quality foods at home. Strategies are needed to sustain cooking and eating at home as commitments and activities resume. Cooking programs may be an effective strategy to support adults in knowledge, skills, and confidence to continue, begin, or expand their at-home cooking [57,58,59].

Participants in the current study also reported engaging in outdoor activities such as gardening as a hobby or for food. Similarly, among other US consumers, up to 35% have reported partaking in local food purchasing or home food procurement activities since the start of the pandemic [60,61,62]. As participants spent more time at home, they stated there was increased interest in gardening among themselves and others. A local community garden saw increased participation from the community at the start of the pandemic. However, participants expressed that, as the pandemic continued in Laurel County, gardening has slowed as businesses reopened, reducing time to care for gardens. Understanding current gardening knowledge within the community, and the motivation or apprehension to garden personally, could be valuable in developing potential strategies for improving home environment access to foods such as fresh produce and enhancing skills and self-efficacy surrounding gardening for food.

Our results exhibit the complex issues surrounding the food environment in Laurel County and the importance of improving existing frameworks such that they can support healthy eating. While most changes in the food environment were viewed quite favorably, it is not clear whether these changes will exacerbate inequities in food access and quality of food intake. For example, it is possible that those of a higher socioeconomic status could take advantage of online food ordering through more reliable internet access and meeting required minimum purchases and delivery costs. Those of lower socioeconomic status or who live in more remote locations may have less reliable internet or fewer food businesses that could sustain financial challenges related to the pandemic [63]. For those spending more consistent time at home, expanding skills surrounding the growing and cooking of foods expanded; however, as businesses return toward normal operations, lifestyles are beginning to return toward pre-COVID habits and behaviors. Moreover, the long-term impacts of COVID-19 on the food environment are yet to be seen. Understanding the shifts in the food environment, and evaluating their efficiency in improving food access, can inform supplementary adaptations to the food system that could be beneficial as we move forward through the pandemic and postpandemic alike [41].

### Limitations

Our study is not without limitations. Community participants were self-selected to participate through Extension-led recruitment and were not randomly selected. Therefore, responses that were received, particularly surrounding changes in community programs, may have been skewed due to increased knowledge of Extension programming in the county.

Likewise, generalizability to other rural populations is limited, as our cohort was a small sample size among a singular rural community. As we were intentional to require minimal inclusion criteria, to obtain feedback from a wide audience, this may have led to a lack of information collected from those most at risk of structural vulnerabilities. Due to our purposive sampling, we are unable to determine external validity and may not have had robust representation from individuals experiencing food insecurity. Conducting an objective food environment assessment that includes robust mixed-methods data from lower socioeconomic status rural community members can assist with comprehensively understanding the facilitators and barriers to accessing food when utilizing governmental assistance benefits (e.g., SNAP) and living in geographically isolated areas that delivery is unavailable. Finally, as these data were collected in part of a larger study, future work should consider more robust mixed-methods questioning of COVID-related food environmental influences.

Despite these limitations, the current study captured previously undocumented experiences of individuals residing in one rural Appalachian community during COVID-19 as it relates to the food environment. This novel feedback lends further insight into the powers of a changing food environment in a rural area and influential resources in the community that are, or were, beneficial to healthful living.

## 5. Conclusions

Though rural communities faced considerable challenges during the COVID-19 pandemic, in part, due to gaps in the existing infrastructure and loss of pre-existing resources, the current study also illustrates the complexity of changes during the pandemic in one rural Appalachian area. In our studied community, loss of civic programs and closure of organizations and worksites left many in financial crisis and ultimately lacking food access. In response, resources such as free meal boxes and the expansion of food banks provided some relief to the community. Newer resources, such as DoorDash food delivery services, also made their way to the county studied as a result of the pandemic; however, areas of service still failed to reach many geographically isolated dwellers.

Understanding shifts in the food environment in response to a pandemic has the potential to influence emergency preparedness in rural communities, especially for those previously vulnerable prepandemic. Through a community-informed approach, the current study identified a range of issues and resources related to the food environment in one rural Appalachian county. Preliminary findings suggest potential shorter-term solutions for Laurel County to include reinitiating access to community education resources through different or multiple delivery mechanisms, programs to support cooking at home, or the promotion of knowledge and self-efficacy around gardening for food. However, systemic issues such as inequitable access to food will require more objective assessments with a representative sample of the community to develop longer-term plans for addressing community food security after the pandemic.

## Figures and Tables

**Table 1:** Focus group questions.

What comes to mind when you hear the phrase “healthy eating?” What sounds “good” about healthy eating? What concerns you about that phrase?
2. Where can you go in your community to find healthy foods? Is it easy to find healthy foods around here? Are fruits and vegetables easy to find in these places?
3. What are some things in this community that help you eat a healthier diet? Are there good sources of information about eating healthily? What are some other resources available to people in Laurel County that can make it easier for them to eat healthier diets? Do you think people in the community value healthy eating? What makes you say that?
4. How many people in your community grow their own food in a home garden? Why/why not?
5. People sometimes go to different places to get enough food to go around when they are running short of money. What types of places do people in your community go to for emergency food and how often? Which of these places works the best to provide food? Why?
6. Can you talk about how the coronavirus pandemic may have changed how people get food in your community?
7. There are many types of programs and initiatives which have helped communities address healthy food issues and diet. We’d like your help in understanding the best approaches to this challenge in your community. One approach focuses on education, often teaching cooking and food budgeting skills. A second approach focuses on making healthy food options available in more places. A final approach relies on persuasion, using marketing and advertising to encourage and facilitate healthy eating.
8. If the goal is to get as many people in Laurel County to improve their diets as possible, what do you see as the strengths of each of these approaches? Education Availability Promotion
9. If the goal is to get as many people in Laurel County to improve their diets as possible, what do you see as the weaknesses of each of these approaches? Education Availability Promotion
10. Of all the things we have talked about today, what is the most important to you? Is this different to what you think is important to the overall community?
11. Of all the things we have talked about today, what do you feel is the most attainable yet urgent to address in the next 5 years?

**Table 2:** Changes in food environment identified by focus group participants in Laurel County, Kentucky, April 2021.

Modifications of Community Food and Nutrition Resources	
Loss of Pre-Existing Nutrition and Health Education Resources	“I did go to classes at the Extension office, but since COVID, they haven’t had those and we used to have them come in our church once a month, and they would do a class on healthy eating and give out prizes and stuff like that.”
	“But they [Laurel Co. Public Library] don’t have such activities yet resumed, because of the pandemic but usually they offer all these courses.”
New or Expanded Emergency Food Assistance	“Well, I know that there are a lot of churches and organizations that are offering like the food boxes.”
	“I know that they set up like at North Laurel High School one day, and it was just, you know, you drive through and get a box.”
**Expansion and Utilization of Online Food Ordering**	
Grocery and Supermarket	“But even, like, grocery delivery from Walmart and Kroger and that kind of thing, you know, people who probably would never have used online grocery ordering and delivery are now using that.”
	“I try to do our grocery orders as part of a pickup so that if I’m in, you know, if I do go to the store, I’m not in there for very long, and honestly, I think it’s something that I’m going to continue to do after, you know, things hopefully get back to whatever the new normal looks like. Because it’s just, you know, more convenient and time saving measure and honestly, I think you spend less too, because you know exactly what you want to get and you put that in there, and you don’t get tempted by you know the displays or anything like that.”
DoorDash/Food Delivery	“I do think there’s a lot more utilizing like online purchasing and DoorDash like pre coronavirus there wasn’t DoorDash in Laurel County, and now there is so that’s different.”
	“We’ve never had any delivery, like the except like pizza, but nothing like even our little, I mean, about all of our mom-and-pop places now even has DoorDash you can have your desserts delivered or wherever you need them now instead.”
Curbside Pick-Up	“I do the curbside because the drive thru is wrapped around. But my [phone] app didn’t work so, I had to get back in that line and go through the drive thru and wait 20 min to get to the door.”
	“Well, for me, it’s pickup. I still live far enough that nobody’s dashing to my door or anything like that.”
**Implications of the Home Food Environment**	
Spending More Time at Home	“I think people were trying to eat more at home.”
	“Kind of for us, you know, really the pandemic has kind of helped with that a little bit because we were so busy before. That we were doing a lot of eating out and not a lot of thinking about, you know, meals at home and planning that. And so, I think that’s one of the positives that’s come out of the pandemic for our family is, you know, being eating at home more, and making better choices, because we’re not doing the fast food and eating out because we’ve got five sporting events this week or whatever.”
Produce Gardening	“I think that was something from the pandemic that a lot of people had gardens last year that hadn’t had gardens in the past, or you know, so I think that’s something else that comes from it there came from it that was positive.”
	“This year we started a garden. There was a program through one of the schools and they gave kids seeds and Jiffy Pellets to put the seeds in. I mean it was so much easier than I thought.”

## Data Availability

Data is available from the PI upon request.

## Supplementary Materials

The following are available online at https://www.mdpi.com/article/10.3390/nu13113929/s1, File S1: Additional quotes by main themes.
