# Supplementary material for: Losses, Gains, and Changes to the Food Environment in a Rural Kentucky County during the COVID-19 Pandemic
Source: Nutrients. 2021 Nov 3;13(11):3929. doi: 10.3390/nu13113929 (PMC8617704; doi:10.3390/nu13113929)
Supplement: Supplementary file 1 [file nutrients-13-03929-s001.zip › nutrients-1398443-supplementary.pdf]

Supplemental Materials

File S1 Additional quotes by main themes

Modifications of Community Food and Nutrition Resources

[Laurel County Public Library] has offered a lot of classes for nutrition and health, and they also invited specialist on diabetes because people would come, and they made presentations about diabetes, and it was like a whole series that would last for several weeks to educate community, who is having diabetes, how to eat properly or what they can do to control the issue with everything else.

If you ever want to hear about people that need food during the pandemic, [local church] had to have the police out there, and they had to close down the road, because there were thousands of people that try to get food that day, and they ran out. And they gave away decent food, because I know the person that was in charge of it, and they brought in food from [local food pantry]. And they lined up there in the gymnasium with them with all of that food that was decent food to be given away, and cars pulled up, thousands of cars, so many cars that, like, I say the police had to end up closing that the street, so people couldn't get down any longer.

I have two uncles who work for a dairy company, and, like, part of the stimulus or part of, you know, the kind of economic recovery, they got contracts with the government, and so they provide like boxes that have milk and cheese and those kinds of things and so like churches or different organizations can contact them and they'll be able to get like a tractor trailer load of those that they can pass out.

Expansion and Utilization of Online Food Ordering.

I don't think, since COVID hit, that you could even go to KFC. KFC is a least an hour wait in the drive thru. People are eating out. They're getting in the drive thru lines, and if they can't go in, they're getting in the line.

The problem I find, for me, with that is I'm particular about picking out my vegetables and fruit because they are a little more costly, and the people who pack that stuff just want to get it done. I have not really liked it for that reason.

Implications of the Home Food Environment

A lot of you know, financial issues and things like that, so you know people that lost their job or were out of work temporarily might have maybe even had the opposite, they may have better been able to only you know, only been able to get McDonald's or you know bread and Bologna or something like that.

To me, though, one of the biggest resources we have is each other. You know, I am often finding myself in the summer, going over to my grandmother's house and seeing a big bag of tomatoes or zucchini or something that you know, a friend who gardens leaves. Because we know a number of people who garden in abundance and you know, so there is a lot of that trading in can sort of thing that comes about, you know, with just the people in that sense.

We have the [community center] ...they do a wonderful job of helping the community...a lot of them are full of food, maybe because of stimulus. You can see what's back in there, they do have a lot of extra food. Families will come in. They do have a lot of other stuff, furniture, etc. Less families are coming in probably because of the money they're getting.
